# Mechanisms and recent advances in non-coding RNAs and RNA modifications in antiplatelet drug resistance

**DOI:** 10.3389/fgene.2025.1618105

**Published:** 2025-09-08

**Authors:** Ping Ni, Kejie Chen, Jing Xiang, Haifeng Shao, Xiaoling Chen, Qiao Chen, Lingling Wang, Junli Hao, Xinyi Huang, Qing Cao, Yali Yang, Quandan Tan, Jie Yang, Suping Li

**Affiliations:** ^1^ School of Medicine, University of Electronic Science and Technology of China, Chengdu, China; ^2^ Department of Neurology, Sichuan Provincial People’s Hospital, University of Electronic Science and Technology of China, Chengdu, China; ^3^ School of Public Health, Chengdu Medical College, Chengdu, China; ^4^ School of Medical and Life Sciences, Chengdu University of Traditional Chinese Medicine Chengdu, Chengdu, China; ^5^ School of Clinical Medicine, Southwest Medical University, Luzhou, China; ^6^ School of Comprehensive Health Management, Xihua University, Chengdu, China; ^7^ School of Biomedical Sciences and Technology, Chengdu Medical College, Chengdu, China; ^8^ Department of Neurology, The First Affiliated Hospital of Chengdu Medical College, Chengdu, China

**Keywords:** cardiovascular diseases, cerebrovascular disease, antiplatelet drug resistance, non-coding RNAs, RNA modifications

## Abstract

The high incidence and mortality rates of cardiovascular and cerebrovascular diseases make them a significant global health challenge. Antiplatelet drugs play a central role in the prevention and treatment of these diseases. Despite the wide range of available antiplatelet drugs, antiplatelet drug resistance is not rare. So optimizing drug use through personalized treatment strategies to achieve maximum therapeutic benefit remains a major challenge in clinical practice. Non-coding RNAs, including microRNAs (miRNAs), long non-coding RNAs (lncRNAs), and circular RNAs (circRNAs), have made significant progress in understanding their regulatory roles in drug resistance, becoming a frontier area of current research. In addition to the regulatory functions of non-coding RNAs, emerging studies have highlighted the role of RNA modifications, such as N6-methyladenosine (m6A), in the regulation of gene expression and cellular processes involved in antiplatelet drug resistance. These modifications contribute to the stability, splicing, and translation of RNA, further influencing their roles in drug resistance mechanisms. In recent years, significant progress has been made in the research of non-coding RNAs and RNA modifications, revealing their crucial roles in the mechanisms of antiplatelet drug resistance. This review focuses on the latest advancements in non-coding RNA research related to antiplatelet drug resistance and explores the emerging field of RNA modifications. It analyzes potential underlying mechanisms and discusses future research directions, aiming to provide new theoretical support and research perspectives for personalized precision antiplatelet.

## Graphical Abstract

**Figure:**
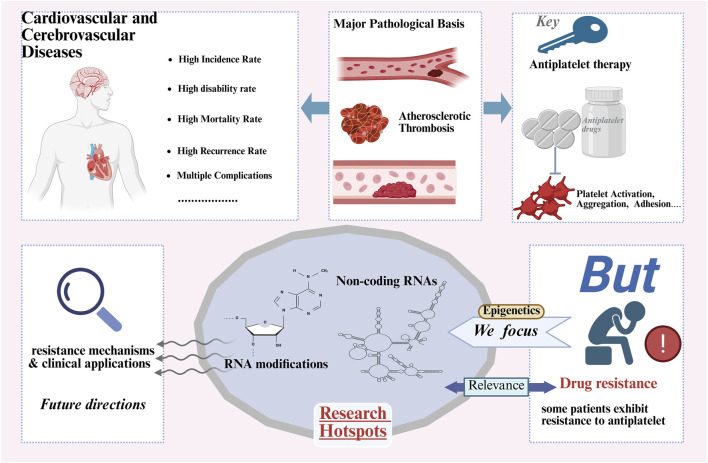
Antiplatelet drugs are key medications in the prevention and treatment of cardiovascular and cerebrovascular diseases, and the issue of drug resistance is receiving increasing attention. Epigenetics, such as non-coding RNAs and RNA modifications (especially m6A), as emerging fields, are gradually playing an important role in antiplatelet drug resistance. This review summarizes the mechanisms of non-coding RNAs and RNA modifications in antiplatelet drug resistance and the related research progress.

## 1 Introduction

Recent global disease burden data indicates that cardiovascular and cerebrovascular diseases pose a heavy burden. These diseases not only lead to increased mortality rates but also result in high disability rates and disease burden, severely affecting public health and quality of life (Li et al., 2023; Li et al., 2024). Antiplatelet drugs are central to the prevention and treatment of cardiovascular and cerebrovascular diseases by inhibiting platelet aggregation, playing a crucial role in the prevention and treatment of cardiovascular and cerebrovascular diseases (Stanger et al., 2023; Greco et al., 2023; Kamarova et al., 2022). However, due to individual differences and the complexity of molecular mechanisms, these drugs may encounter varying degrees of resistance in clinical applications. Notably, approximately 20% of patients exhibit dual high on-treatment platelet reactivity to aspirin and clopidogrel (Breet et al., 2011). Aspirin resistance occurs in up to 60% of cases (Sambu et al., 2013), while clopidogrel resistance reaches 40% (Angiolillo et al., 2007), both correlating with increased risks of atherothrombotic events. Approximately 10%–30% of patients with antiplatelet resistance experience ischemic events (Udell et al., 2016; Li et al., 2012; Reny et al., 2012). A prospective multicenter registry study demonstrated significantly elevated stent thrombosis risk, particularly showing 1.49-fold increased risk in clopidogrel high-responders (Stone et al., 2013). Overall, patients with antiplatelet resistance face 2- to 3-fold higher cardiovascular event risks compared to normal responders (Gum et al., 2003; Gurbel et al., 2003).

Antiplatelet drug resistance currently lacks a standardized definition but is broadly categorized into laboratory resistance and clinical resistance. This refers to the phenomenon where patients experience thrombotic events or demonstrate laboratory-confirmed failure of platelet function inhibition despite receiving standard antiplatelet therapy (Hankey and Eikelboom, 2006; Floyd and Ferro, 2015). Based on mechanistic characteristics, it can be divided into two types: primary resistance and secondary resistance. Primary resistance stems from the patient’s inherent inherited pharmacogenetic abnormalities, including genetic polymorphisms of drug-metabolizing enzymes (such as CYP2C19 loss-of-function alleles) or target receptor variations (like P2Y12 receptor polymorphisms), resulting in the inability of the drug to achieve the expected antiplatelet effect from the outset of therapy (Hou, 2024; Fitzgerald and Pirmohamed, 2011; Pereira et al., 2019; Akkaif et al., 2021). Secondary resistance occurs after initially effective treatment and is triggered by acquired factors, commonly including drug-drug interactions (e.g., proton pump inhibitors competitively inhibiting clopidogrel metabolism), disease-related enhancement of platelet activation (e.g., in inflammatory conditions such as diabetes), accelerated platelet turnover, or poor patient adherence (Hankey and Eikelboom, 2006; Huang et al., 2025; Kaur et al., 2018). Resistance not only leads to reduced therapeutic efficacy but also increases the risk of vascular events, thereby affecting the overall prognosis of patients (Hankey and Eikelboom, 2006; Spanos et al., 2017; Ball, 2009). The mechanisms of antiplatelet drug resistance are complex and are often closely related to factors such as variations in drug-metabolizing enzymes, mutations in target proteins, and epigenetic regulation (Fitzgerald and Pirmohamed, 2011; Pereira et al., 2019; Kim et al., 2024). Traditional research has primarily focused on the genetic factors of drug resistance, with relatively few exploration into the epigenetic mechanisms, particularly non-coding RNAs.

Non-coding RNAs refer to RNA molecules that do not encode proteins, primarily including microRNAs (miRNAs), long non-coding RNAs (lncRNAs), and circular RNAs (circRNAs). These non-coding RNAs influence various biological processes by regulating gene expression, cellular physiological processes, and other biological functions (Chen and Kim, 2024). miRNAs, small RNA molecules of approximately 22 nucleotides, play a key role in regulating complex genetic networks and cellular signaling pathways (Bushati and Cohen, 2007; Bartel, 2004; Lee et al., 1993). In addition to being potential biomarkers and diagnostic tools, miRNAs have shown great promise in disease treatment (Diener et al., 2022; MicroRNAs, 2017). Studies have demonstrated the regulatory role of miRNAs in platelet function. For instance, the effector complex formed by miR-223 and Ago2 can specifically target the purinergic receptor P2Y12, which is involved in platelet aggregation, thereby modulating platelet activation (Landry et al., 2009). Existing studies have demonstrated that miRNAs play an important role in regulating platelet function and reactivity, as well as in the mechanisms of antiplatelet drug resistance (Stojkovic et al., 2019; Singh et al., 2021; Willeit et al., 2013). lncRNAs, typically composed of hundreds to thousands of nucleotides, are widely involved in transcriptional regulation, epigenetic regulation, translation, and other cellular processes (Bridges et al., 2021; Hangauer et al., 2013). Research has revealed that MT1P3 upregulates P2Y12 by sponging miR-126, thereby promoting platelet hyperreactivity in diabetes (Zhou et al., 2019). The role of lncRNAs in antiplatelet drug resistance has gradually become a research hotspot (Wang et al., 2020). circRNAs are RNA molecules with a closed-loop structure, primarily regulate gene expression through functions such as acting as miRNA sponges and regulating protein translation (Zhou WY. et al., 2020). CircRNAs are abundantly expressed in human platelets (Alhasan et al., 2016), Platelet-derived circRNAs can interact with protein complexes of varying sizes, as exemplified by the platelet-specific circRNA Plt-circR4 (Preußer et al., 2018). Although research on circRNAs in antiplatelet drug resistance is still in its early stages, existing findings suggest that circRNAs hold significant potential in resistance mechanisms (Xu et al., 2025). Recent studies have also suggested that RNA modifications, such as N6-methyladenosine (m6A), may affect the functional roles of circRNAs and other non-coding RNAs in modulating gene expression and contributing to antiplatelet drug resistance (Yu et al., 2023). m6A modification, a prevalent and dynamic RNA modification, has been implicated in the regulation of RNA stability, splicing, translation, and the degradation of non-coding RNAs (Yue et al., 2019; Lence et al., 2017), thus influencing their role in drug resistance mechanisms (Liu and Pan, 2016). However, there are currently no reported studies on the roles of other RNA epigenetic modifications (such as m5C, Ψ, etc.) in antiplatelet drug resistance, and this field urgently requires further investigation.

This article primarily reviews the role of non-coding RNAs in the resistance to antiplatelet drugs, exploring their potential molecular mechanisms and providing an outlook on future research directions. In addition, the article also discusses the emerging field of RNA modifications, with a particular focus on m6A modification, and its potential impact on antiplatelet drug resistance.

## 2 Non-coding RNAs and RNA modifications in aspirin resistance

Aspirin, as a core drug for the prevention and treatment of cardiovascular and cerebrovascular diseases, has been consistently recommended as a Class I medication in both domestic and international guidelines (Dawson et al., 2022; Abdelaziz et al., 2019; Li and Zhao, 2024). However, approximately 20%–60% of patients develop resistance to aspirin, which not only weakens its therapeutic effect but also presents a significant challenge for clinicians when formulating treatment plans (Sambu et al., 2013; Fiolaki et al., 2017; Khan et al., 2022). Previous studies have revealed that the mechanisms of aspirin resistance are primarily linked to genetic factors, drug interactions, patient adherence, and inflammatory responses (Hankey and Eikelboom, 2006; Fitzgerald and Pirmohamed, 2011; Floyd and Ferro, 2014). With the rapid development of high-throughput genomics and chip technologies, scientists have gradually recognized the potential role of non-coding RNAs in the mechanisms of aspirin resistance. Recent research has also gradually unveiled the complex mechanisms of non-coding RNAs in aspirin resistance (Table 1), providing new directions for the development of novel biomarkers and drug intervention strategies.

**TABLE 1:** Research Summary: mechanisms of Non-coding RNAs and RNA Modifications in aspirin resistance and platelet reactivity regulation.

Non-coding RNAs	Study population	Drug	Platelet function testing and definition of resistance	Potential targets	Findings/Conclusions	Ref.
miR-19b-1-5p	ACS Patients (n = 945)	Aspirin	Multiplate Analyzer measurement; ASPItest ≥30 U	GUCY1A3, NOS3, PDE5	Low expression of miR-19b-1-5p is associated with persistent platelet aggregation during aspirin therapy	Singh et al. (2021)
miR-34b-3p	CAD patients (n = 113)	Aspirin	Light transmission aggregation (LTA); high reactivity refers to the lowest quartile of platelet aggregation	TBXAS1	miR-34b-3p may regulate platelet function and aspirin response	Liu et al. (2019)
miR-135a-5p, miR-204-5p	Patients with symptomatic atherosclerotic thrombotic disease (n = 110)	Aspirin	Light transmission aggregation (LTA) measurement; PR index is at the extreme values	THBS1, CDC42, CORO1C, SPTBN1, TPM3, GTPBP2, MAPRE2	miR-135a-5p and miR-204-5p are associated with platelet reactivity	Zufferey et al. (2016)
miR-92a	Patients with intermittent claudication (n = 209)	Aspirin	Multiplate Analyzer measurement; ASPItest ≥30 U	NA	miR-92a is associated with aspirin resistance	Binderup et al. (2019)
miR-126	CAD patients (n = 118)	Aspirin	Automated platelet aggregation analyzer; AA-induced platelet aggregation rate ≥20%	VEGF, COX-2	Urinary miR-126 can serve as an independent risk factor for aspirin resistance	Fan et al. (2016)
miR-126	CAD patients (n = 106)	Aspirin	Automated platelet aggregation analyzer; AA-induced platelet aggregation rate ≥20%	NA	Platelet miR-126 is closely related to aspirin resistance	Liu et al. (2017)
miR-223	AIS patients (58)	Aspirin/Aspirin + Clopidogrel	Whole blood impedance method; ADP-induced platelet aggregation rate ≥70% or AA-induced ≥20%	NA	miR-223 and aspirin resistance in patients with AIS	Chen et al. (2021)
lncRNA H19	AIS patients (n = 150)	Aspirin	ELISA kit measuring the level of 11dhTXA2 in urine; urine 11dhTXA2/creatinine ratio >1,500 pg/mg	8-iso-PGF2	H19 long non-coding RNA is closely associated with aspirin resistance	Wang et al. (2020)
RNA N6-methyladenosine methylation	Elderly patients requiring primary or secondary prevention (n = 34)	Aspirin	Turbidimetric assay; platelet aggregation rate <7%	NA	RNA m6A methylation level is elevated in elderly patients with low aspirin responsiveness	Zhang et al. (2023)

1CORO1C, Coronin 1C; SPTBN1, Spectrin Beta Non-Erythrocytic 1; TPM3, Tropomyosin 3; GTPBP2, GTP Binding Protein 2; MAPRE2, Microtubule Associated Protein RP/EB Family Member 2; PR Index, Platelet Reactivity Index; NA, means no.

### 2.1 The role of miRNAs in aspirin resistance

Research indicates that miRNAs play a key role in platelet function and the mechanisms of aspirin resistance by regulating gene expression. Multiple studies have confirmed that specific miRNAs can serve as important biomarkers for aspirin resistance. Among them, low expression of miR-92a combined with platelet distribution width (PDW) shows high sensitivity and specificity; plasma miR-92a levels are significantly higher in aspirin-resistant patients compared to aspirin-sensitive patients (Binderup et al., 2016; Binderup et al., 2019). Additionally, downregulation of miR-19b-1-5p is closely associated with aspirin resistance and an increased risk of major adverse cardiovascular and cerebrovascular events (MACCE) in patients with acute coronary syndrome (ACS) (Singh et al., 2021; Kok et al., 2016). In patients with acute ischemic stroke (AIS), miR-223 has also been shown to be significantly associated with aspirin resistance (Chen et al., 2021). From a mechanistic perspective, miR-135a-5p and miR-204-5p affect the aspirin response by regulating a gene network including thrombospondin-1 (THBS1) and cell division cycle protein 42 (CDC42) (Zufferey et al., 2016). MiR-34b-3p regulates platelet function by inhibiting thromboxane A synthase (TBXAS1) and megakaryocyte proliferation (Liu et al., 2019). MiR-126 is involved in resistance formation by promoting platelet activation and aggregation, and its urinary level has been identified as an independent risk factor for aspirin resistance (Liu et al., 2017; Fan et al., 2016). Notably, long-term aspirin treatment can lead to downregulation of miR-26b expression in platelets, which in turn upregulates multidrug resistance protein 4 (MRP4) expression, enhancing platelet residual reactivity (La et al., 2018; Massimi et al., 2016). This provides a new basis for personalized adjustments in clinical long-term medication. These findings not only reveal the core regulatory role of miRNAs in aspirin resistance but also demonstrate their important clinical value in personalized treatment and resistance monitoring, laying the theoretical foundation for the development of precise antiplatelet strategies.

### 2.2 LncRNA and aspirin resistance

Large-scale analyses have revealed the complex expression profiles of lncRNAs in platelets and explored their correlation with platelet reactivity, suggesting that lncRNAs may serve as novel platelet function regulators (Sun et al., 2022). In a study of patients with AIS, the polymorphism of the H19 gene was closely associated with susceptibility in this population. It was found that H19 lncRNA induces aspirin resistance by promoting the generation of eight-iso-Prostaglandin F2α (8-iso-PGF2) (Wang et al., 2020). These findings further demonstrate the significant role of specific lncRNAs in aspirin resistance. Although current research on lncRNAs in aspirin resistance is limited, ncRNAs provide novel perspectives for future personalized treatment and aspirin resistance prediction.

### 2.3 CircRNA and aspirin resistance

Current research on the molecular mechanisms by which circRNAs regulate aspirin resistance remains an unexplored field. Notably, as competing endogenous RNAs (ceRNAs), circRNAs can specifically sequester microRNAs through their “molecular sponge” effect, thereby relieving miRNA-mediated suppression of target mRNAs (Chen and Lu, 2021; Panda, 2018; Liu et al., 2018). Studies have revealed significant correlations between plasma circRNA expression profiles and platelet activity in heart failure patients (Sun et al., 2020). Building upon the ceRNA regulatory network theory, future integration of multi-omics technologies with network pharmacology approaches may provide novel targeted therapeutic strategies for personalized antiplatelet therapy.

### 2.4 RNA modifications and aspirin resistance

Emerging evidence suggests that m6A RNA methylation may regulate key genes in platelet activation pathways (e.g., PIK3R5, PLCG2) through post-transcriptional modifications, including mRNA stability, splicing efficiency, and translational dynamics, thereby modulating platelet activation thresholds and aggregation capacity (Xu et al., 2021). Furthermore, studies indicate that alterations in m6A methylation can influence platelet function and subsequently affect aspirin responsiveness in elderly patients (Zhang et al., 2023). Such methylation modifications may contribute to interindividual variability in drug response among aging populations, ultimately impacting aspirin’s therapeutic efficacy. Currently, no direct evidence supports associations between other RNA modifications (e.g., m^5^C, ac^4^C, Ψ) and platelet function. Although research on RNA methylation remains limited, further investigation in this field may provide novel mechanistic insights into platelet regulation and facilitate the development of personalized antiplatelet therapies.

## 3 Non-coding RNAs and clopidogrel resistance

Clopidogrel is a commonly used antiplatelet drug for cardiovascular and cerebrovascular diseases, known for its high safety and effectiveness. It significantly reduces the risk of cardiovascular and cerebrovascular events and plays an important role in prevention and treatment (Gimbel et al., 2020; Valgimigli et al., 2024; Chen et al., 2024). However, approximately 30%–45% of patients may develop clopidogrel resistance (Fiolaki et al., 2017; Ray, 2014). Clopidogrel resistance remains an important challenge in clinical treatment, particularly closely related to individual differences in platelet reactivity. The mechanisms of clopidogrel resistance are complex, involving genetic factors, drug interactions, clinical factors, and other aspects (Pereira et al., 2019; Nguyen et al., 2005). In recent years, significant progress has been made in research on the role of non-coding RNAs in the response to clopidogrel antiplatelet therapy (Table 2). Increasing evidence suggests that changes in the expression of non-coding RNAs during clopidogrel treatment have a profound impact on resistance, making it a key focus of research on this issue.

**TABLE 2:** Research synthesis: regulatory roles of non-coding RNAs and RNA Modifications in clopidogrel resistance and platelet reactivity.

Non-coding RNAs	Study population	Drugs	Platelet function testing and definition of resistance	Potential targets	Findings/Conclusions	Ref.
miR-199a-5p	CAD patients (n = 508)	Clopidogrel+ Aspirin	Flow cytometry detection; HTPR is defined as PRI >50%	VASP	Decreased levels of miR-199a-5p are associated with increased platelet reactivity after clopidogrel treatment	Hu et al. (2023)
miR-223	NSTE-ACS patients (n = 33)	Clopidogrel + Aspirin	Flow cytometry measured PRI and light transmission aggregation measured PAG; PRI >56.5%, PAG >43%	P2Y12	Low expression of miR-223 is associated with clopidogrel resistance	Shi et al. (2013)
MiR-223 , miR-126	STEMI patients (n = 120)	Clopidogrel + Aspirin	VerifyNow analyzer; PRU >208	NA	miR-223 and miR-126 play a role in dual antiplatelet therapy resistance	Li et al. (2021)
miRNA-142-3p, miRNA-24-3p, miRNA-411-3p	CAD patients (n = 66)	Clopidogrel + Aspirin	VerifyNow analyzer; Platelet aggregation (IPA) < 30%	NA	These three miRNAs may be potential biomarkers for clopidogrel resistance	Lin et al. (2021)
miR-223, miR-221, miR-21	ACS patients (n = 272)	Clopidogrel + Aspirin	Light transmission aggregation (LTA); RI < 10%	P2Y12	These three miRNAs play a role in clopidogrel resistance	Peng et al. (2017)
miR-223, miR-21	CAD patients undergoing PCI(n = 119)	Clopidogrel + Aspirin	Thromboelastography (TEG5000); Platelet inhibition rate <50%	NA	miR-223 and miR-21 are associated with clopidogrel resistance	Guo et al. (2020)
miR-126, miR-130a, miR-27a, miR-106a, miR-21 and miR-142	CAD patients (n = 1,230)	Clopidogrel + Aspirin	VerifyNow analyzer; PRUs >208	NA	These six miRNAs are associated with platelet aggregation in patients treated with clopidogrel	Tang et al. (2019)
miR-1343-3p, hsa-miR-6783-3p	PCI or ACS patients (n = 292)	Clopidogrel + Aspirin/Clopidogrel	Platelet aggregation assessed by Chronolog Lumi-Aggregometer and its AggroLink software; ΔA < 10%	CYP2C19	mirSNPs regulate CYP2C19 gene expression through miRNAs, affecting clopidogrel drug response	Sharma et al. (2020)
miR-107	ACS patients (n = 50)	Clopidogrel + Aspirin	VerifyNow analyzer; PRU ≥300	P2Y12	Platelet miR-107 is associated with clopidogrel resistance	Zhang et al. (2022)
miR-126	ACS patients (n = 364)	Clopidogrel + Aspirin	Thromboelastography (TEG); TEG MAADP >47 mm, ADP inhibition rate <30%	P2Y12	miR-126 may affect the reactivity and efficacy of clopidogrel antiplatelet therapy	Zhou et al. (2021)
miR-223, miR-126, miR-150	ACS patients (n = 430)	Clopidogrel + Aspirin	TEG; the top 10 platelet reactivity values are considered high platelet reactivity	P2Y12, ADAM9 , PI3KR2	These three miRNAs may be associated with changes in clopidogrel’s antiplatelet response	Liu et al. (2020)
miRNA-26a	ACS patients undergoing PCI(n = 201)	Clopidogrel + Aspirin	Light transmission aggregation (LTA); Platelet aggregation >59%	VASP mRNA	Upregulation of miRNA-26a is associated with clopidogrel resistance	Giantini et al. (2022)
lncRNA (NONHSAT083775.2, NONHSAT 107804.2, NONHSATl33455.2)	CAD patients (n = 136)	Clopidogrel	Whole blood electrical impedance method; Platelet aggregation rate ≥10 Ω	NA	The differential expression of these three lncRNAs is associated with clopidogrel resistance	Xie et al. (2019)
hsa_circ_0076957 hsa_circ_0057714	Patients with stable CAD (n = 50)	Clopidogrel + Aspirin	VerifyNow analyzer; PRU >240	COL19A1	hsa_circ_0057714 and hsa_circ_0076957 as novel biomarkers for clopidogrel resistance	Xu et al. (2025)
				AOX1		
N6-methyladenosine	CAD Patients (n = 46)	Clopidogrel + Aspirin	VerifyNow analyzer; PRU >240	NA	Revealed the m6A transcriptomic profile of clopidogrel resistance	Yu et al. (2023)
N6-methyladenosine	IS Patients (n = 10)	Clopidogrel	Automated Platelet Aggregation Analyzer (PL-12); Platelet aggregation inhibition rate <30%	CYP2C19	METTL3-mediated CYP2C19 mRNA methylation is associated with clopidogrel resistance	Tan et al. (2024)

### 3.1 miRNA and clopidogrel resistance

#### 3.1.1 miR-223 and clopidogrel resistance

Studies have shown that in patients with non-diabetic coronary heart disease and non-ST elevation acute coronary syndrome (NSTE-ACS), miR-223 plays an important role in regulating platelet function by targeting key signaling pathways downstream of the adenosine diphosphate (ADP) receptor (P2Y12), and it can serve as a potential biomarker for predicting clopidogrel resistance (Shi et al., 2013). Meta-analysis further supports this view, revealing that lower plasma levels of miR-223 are independently associated with clopidogrel resistance in Chinese ACS patients (Cheng et al., 2023). In a study on the GAS5 single nucleotide polymorphism (SNP) rs55829688, GAS5 was found to act as a competitive endogenous RNA for miR-223-3p, regulating the expression of the P2Y12 receptor, which in turn affects the response of coronary heart disease patients with poor metabolic genotypes of CYP2C19 to clopidogrel. This mechanism highlights the potential role of GAS5 in antiplatelet therapy, particularly in regulating clopidogrel response (Liu et al., 2021). Additionally, research has found that miR-223 and miR-21 are associated with clopidogrel resistance, especially in coronary heart disease patients undergoing percutaneous coronary intervention (PCI) (Guo et al., 2020). In summary, miR-223 may serve as a potential biomarker for predicting clopidogrel resistance, helping doctors make early predictions during treatment.

#### 3.1.2 The role of other miRNAs in clopidogrel resistance

In addition to miR-223, several other miRNAs have been implicated in clopidogrel resistance, such as miR-142-3p, miR-24-3p, and miR-411-3p, may play a role in the mechanism of clopidogrel resistance in CAD patients by regulating genes associated with platelet activation (Lin et al., 2021). In CAD patients, miR-199a-5p can inhibit the expression of vasodilator-stimulated phosphoprotein (VASP), and its decreased levels are significantly associated with increased platelet reactivity after clopidogrel treatment (Hu et al., 2023). This finding suggests that miR-199a-5p may play an important regulatory role in antiplatelet therapy for CAD. Studies have also found that miR-107 is involved in the mechanism of clopidogrel resistance after percutaneous coronary intervention PCI by regulating the expression of the P2Y12 receptor (Zhang et al., 2022). The findings underscore the intricate involvement of miRNAs in clopidogrel resistance, which is central to the challenge of individualizing treatment in CAD patients.

#### 3.1.3 miRNA as potential biomarkers for cardiovascular events

Additional research has explored the role of miRNAs as biomarkers for predicting clopidogrel resistance and major cardiovascular events. Changes in plasma miRNAs, such as miR-142, have been proposed as potential biomarkers for predicting major adverse cardiovascular events, especially in patients receiving dual antiplatelet therapy (Tang et al., 2019). Several studies have shown that the expression of miR-26a is related to platelet hyperreactivity, and upregulation of miR-26a is associated with clopidogrel resistance after coronary artery stent implantation (Giantini et al., 2022; Chen et al., 2016). Furthermore, the functional genetic polymorphism rs4636297 of platelet-derived miR-126 may affect the response and efficacy of clopidogrel antiplatelet therapy in ACS patients and is associated with major ischemic events within 1 year (Zhou et al., 2021). miR-223 and miR-126 have been identified as potential predictors of clopidogrel resistance in ST-segment elevation myocardial infarction (STEMI) patients undergoing dual antiplatelet therapy (Li et al., 2021). Other studies have also suggested that platelet-derived miR-223, miR-126, and miR-150 may play an important role in regulating the differential response of ACS patients to clopidogrel antiplatelet therapy (Liu et al., 2020). These findings further underscore the essential role of miRNAs in clopidogrel resistance mechanisms.

#### 3.1.4 Interaction between miRNA and functional genotype in clopidogrel efficacy

The interaction between miRNAs and genetic polymorphisms, particularly the CYP2C19 genotype, provides new insights into understanding the individual differences in clopidogrel response. Current studies indicate that patients carrying mutations in the CYP2C19 gene (such as CYP2C19*2) generally show poor responses to clopidogrel (Pereira et al., 2019; Lee et al., 2022). Specifically, the mirSNP rs4244285, which encodes hsa-miR-1343-3p and hsa-miR-6783-3p, regulates the expression of the CYP2C19 gene, thereby influencing clopidogrel drug response, with the potential to serve as a predictive biomarker, particularly in the Indian population (Sharma et al., 2020). In addition, in ACS patients, miR-223, miR-221, and miR-21 may enhance platelet activation, and in combination with the CYP2C19 genotype, they jointly affect clopidogrel resistance (Peng et al., 2017). Similarly, the miR-605 rs2043556 polymorphism has also attracted attention for its effect on clopidogrel efficacy. miR-605 regulates the expression of the CYP2B6 and P2RY12 genes, affecting the antiplatelet effect of clopidogrel and may serve as a potential biomarker for predicting the risk of cardiovascular events in patients on long-term clopidogrel therapy (Zhou WL. et al., 2020). These studies highlight the interaction between miRNAs and functional genotype.

### 3.2 CircRNA and lncRNA in clopidogrel resistance

Studies have shown that circRNAs and lncRNAs play an important role in clopidogrel resistance. For example, hsa_circ_0076957 and miR-4512 jointly regulate the expression of the COL19A1 gene, which may affect platelet reactivity and clopidogrel efficacy (Xu et al., 2025). In CAD patients, differential expression of lncRNAs in clopidogrel resistance reveals new molecular mechanisms. Although the specific mechanisms are still under investigation, certain lncRNAs, such as the upregulation of NONHSAT083775.2 and NONHSAT107804.2 and the downregulation of NONHSAT133455.2, are believed to be associated with clopidogrel resistance (Xie et al., 2019). Additionally, studies have found that the lncRNA metallothionein pseudogene 1 (MT1P) upregulates miR-126 through a sponge effect, thereby promoting the expression of P2Y12, which may lead to excessive platelet activation (Zhou et al., 2019). These non-coding RNAs contribute to the development of resistance by regulating platelet function and signaling pathways associated with clopidogrel response.

### 3.3 RNA modifications and clopidogrel resistance

m6A methylation is considered to play a crucial role in clopidogrel resistance. Studies targeting CAD patients have revealed m6A transcriptomic features associated with clopidogrel resistance (Yu et al., 2023). Additionally, in ischemic stroke (IS) patients and clopidogrel-resistant animal models, the m6A methyltransferase METTL3 may exacerbate clopidogrel resistance by regulating the methylation of CYP2C19 mRNA (Tan et al., 2024). This finding suggests that epigenetic regulatory mechanisms such as RNA modifications may play a key role in the individual differences in clopidogrel efficacy.

In conclusion, the key roles of non-coding RNAs and RNA modifications in clopidogrel responses provide new research perspectives for a deeper understanding of the molecular mechanisms underlying clopidogrel resistance. These findings not only contribute to advancing the knowledge of clopidogrel resistance mechanisms but also offer important biomarker support for the individualized treatment and clinical management of clopidogrel therapy, thereby providing a solid theoretical foundation for developing more precise therapeutic strategies.

## 4 Non-coding RNAs and ticagrelor tesistance

Ticagrelor is a rapidly absorbed and reversible P2Y12 receptor antagonist, widely used in the treatment of cardiovascular diseases due to its potent antiplatelet effects (Kabil et al., 2022). Ticagrelor is generally considered an effective alternative for patients who do not respond to clopidogrel. Research on ticagrelor shows that for patients with acute coronary syndrome, switching to ticagrelor monotherapy after 3 months of dual antiplatelet therapy significantly reduces the composite risk of major bleeding and cardiovascular events year than 12 months of dual antiplatelet therapy (Kim et al., 2020). However, although ticagrelor resistance is rare, it still occurs in some patients. In a case report, the VerifyNow analyzer detected a lack of response to clopidogrel in a patient, and although the treatment was switched to ticagrelor, platelet inhibition remained suboptimal, leading to adverse events (Kuhn et al., 2024). Another study explored the existence of ticagrelor resistance and proposed management strategies for patients with ticagrelor resistance (Laurent et al., 2022). Currently, research on the role of non-coding RNAs in ticagrelor resistance is limited.

### 4.1 miRNA and ticagrelor resistance

Studies have found that ticagrelor shows more significant efficacy in CAD patients receiving different antiplatelet treatment regimens, and the expression level of miR-365-3p is associated with the response to antiplatelet therapy (Chen et al., 2019). In experimental models, researchers constructed a ticagrelor-resistant platelet inhibition transfection model using the MEG-01 cell line and verified the effect of miR-126-3p on ticagrelor activation inhibition. They demonstrated that miR-126-3p affects ticagrelor’s antiplatelet reactivity by regulating the PI3K-Akt pathway (Wang, 2022). This finding provides new molecular clues for the mechanism of ticagrelor resistance and lays the theoretical foundation for future personalized treatment strategies for drug efficacy.

### 4.2 CircRNA and ticagrelor resistance

Studies have shown that platelet-derived circFAM13B is upregulated in patients with high platelet reactivity (HTPR) and is unrelated to traditional clinical risk factors. It can predict adverse ischemic events in ACS patients after ticagrelor treatment. Bioinformatics analysis suggests that circFAM13B may bind to miR-126, indicating its potential involvement in mechanistic exploration in future studies (Zou et al., 2024). Furthermore, researchers analyzed the global transcriptional effects of ticagrelor on platelets, which helps identify patients who may be adversely affected by ticagrelor treatment, potentially preventing the occurrence of the first arterial thrombotic events (Myers et al., 2024). These findings highlight the importance of circFAM13B as a potential biomarker in ticagrelor resistance, particularly in predicting treatment response and preventing adverse thrombotic events.

Although current research on the role of non-coding RNAs in ticagrelor resistance is limited, with no reports on non-coding RNA fields such as lncRNAs (Table 3), considering the significant effect of ticagrelor in reducing cardiovascular event risk, future studies on its resistance are expected to further optimize clinical applications.

**TABLE 3:** Summary of studies on non-coding RNAs in ticagrelor resistance.

Non-coding RNAs	Study population	Drugs	Platelet function testing and definition of resistance	Potential targets	Ref.
miR-126-3p	ACS patients (n = 129)	Ticagrelor + Aspirin	TEG; Platelet reactivity at extremely high levels	PI3K-Akt pathway	Wang (2022)
circFAM13B	ACS patients (n = 272)	Ticagrelor	TEG; ADP% < 76%	miR-126	Zou et al. (2024)

## 5 Non-coding RNAs and prasugrel resistance

Prasugrel, as another P2Y12 antagonist, has shown similar cardiovascular adverse event rates and bleeding risks compared to ticagrelor in clinical studies (Bundhun et al., 2017). Further research indicates that patients treated with prasugrel exhibit similarities in the levels of various platelet-associated miRNAs and monocyte-platelet aggregate indicators when compared to patients treated with ticagrelor (Stojkovic et al., 2021). In patients receiving increased doses of aspirin and prasugrel, antiplatelet therapy significantly reduced the levels of circulating platelet-derived miRNAs (Willeit et al., 2013). A study on type 2 diabetic patients treated with aspirin, clopidogrel, and prasugrel monotherapy showed that prasugrel effectively suppressed platelet activity and lowered the levels of multiple platelet-associated miRNAs (Parker et al., 2020). These findings suggest that prasugrel may enhance its antiplatelet effect by regulating miRNA levels. In the future, non-coding RNA research based on miRNAs will provide new insights into the exploration of prasugrel resistance mechanisms and promote further development in this field.

## 6 The prospect of noncoding RNA in antiplatelet drug resistance

**FIGURE 1:**
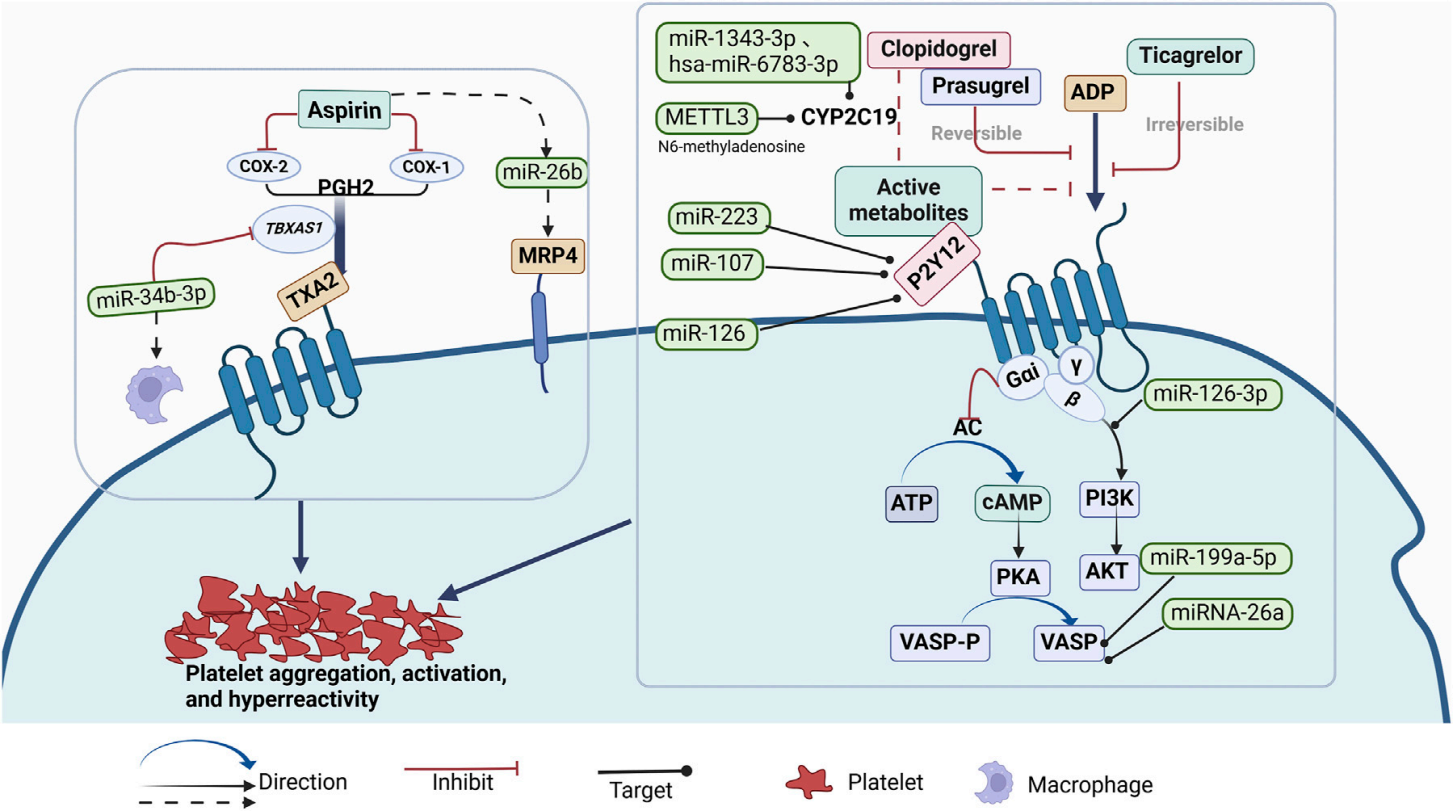
Mechanism of Action of Antiplatelet Drgs and Known miRNAs Regulating the Effects of Antiplatelet Drugs (such as Aspirin, Clopidogrel, Prasugrel, Ticagrelor) on Platelet Aggregation, Activation, and Hyperreactivity Through Multiple Signaling Pathways.

Aspirin and clopidogrel are widely used in the prevention and treatment of cardiovascular and cerebrovascular diseases and have become the core focus of antiplatelet resistance research. Existing studies suggest that non-coding RNAs play a key role in the resistance mechanisms of these two drugs, particularly the influence of platelet-related miRNAs in the formation of drug resistance. Figure 1 illustrates the mechanisms of action of antiplatelet drugs and how miRNAs regulate drug efficacy through multiple signaling pathways. However, research on the relationship between non-coding RNAs and other antiplatelet drugs (such as ticagrelor, prasugrel, etc.) in resistance is still relatively scarce, and some drugs lack related research reports.

**FIGURE 2:**
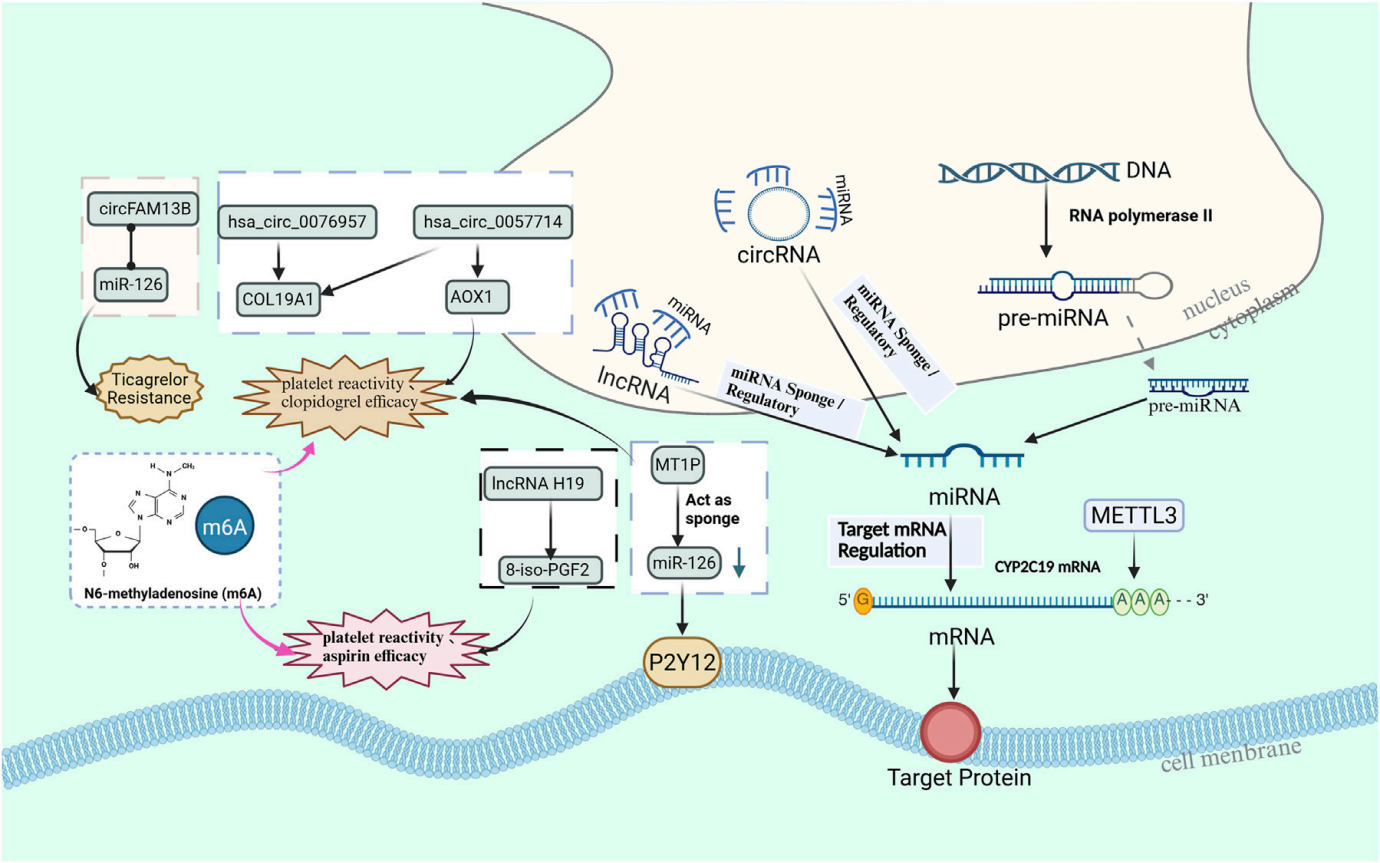
The miRNA sponge mechanism and interactions of non coding RNAs, particularly lncRNAs and circRNAs, associated with antiplatelet resistance and platelet response.

In addition, the role of other non-coding RNAs, such as lncRNA and circRNA, in antiplatelet drug resistance is also receiving increasing attention. Figure 2 further demonstrates how lncRNAs and circRNAs regulate resistance through the sponge mechanism and the known mechanisms of non-coding RNAs and RNA modifications associated with drug resistance. With the expanding application of RNA-based therapies, future research should delve deeper into the role of non-coding RNAs and RNA modification mechanisms in antiplatelet drug resistance, providing a more accurate theoretical foundation and new perspectives for personalized treatment.

## 7 Discussion

This review summarizes the biological relationship between non-coding RNAs and resistance to different antiplatelet drugs, with a focus on analyzing the potential mechanisms of action of different types of non-coding RNAs in antiplatelet drug resistance. Currently, as an important component of epigenetics, the study of non-coding RNAs in antiplatelet drug resistance is still in its early stages, but their crucial regulatory roles have been preliminarily confirmed, especially for miRNAs.

Current research reveals the significant role of non-coding RNAs in the mechanisms of aspirin resistance, particularly the potential regulatory mechanisms of molecules such as miRNAs, lncRNAs, and circRNAs in platelet function and aspirin response. The expression changes of miRNAs such as miR-19b-1-5p, miR-92a, and miR-34b-3p are closely related to aspirin resistance, offering new biomarkers that could aid in early diagnosis and personalized treatment. Furthermore, lncRNAs such as H19 play a role in platelet reactivity and aspirin response, further supporting the potential of non-coding RNAs as predictive tools for resistance. Although research on circRNAs in aspirin resistance is still limited, their unique function in gene regulation makes them a promising area for future research. At the same time, RNA modifications, especially m6A methylation, are also considered key factors influencing platelet function and aspirin efficacy, potentially exhibiting different drug responses, particularly in elderly patients. By considering the interplay between non-coding RNAs and RNA modifications, future studies could provide more precise strategies for personalized treatment.

The significance of non-coding RNAs in the mechanisms of clopidogrel resistance is also highly important. The role of miRNAs in regulating clopidogrel resistance in patients with CAD is an important area of research. In addition to miR-223, several other miRNAs, such as miR-142-3p, miR-24-3p, miR-411-3p, miR-199a-5p, and miR-107, are involved, providing new insights into the molecular mechanisms driving resistance to antiplatelet therapy. Furthermore, the interaction between miRNAs and genetic factors, particularly the CYP2C19 genotype, highlights the complexity of individual differences in clopidogrel response. In addition, circRNAs and lncRNAs regulate platelet function and key signaling pathways, offering new insights into the molecular mechanisms driving resistance. RNA modifications, especially m6A methylation, add another layer of complexity by influencing gene expression and further affecting therapeutic outcomes. As we move towards personalized medicine, non-coding RNAs provide a promising tool to predict which patients will have a poor response to clopidogrel treatment. Their potential as biomarkers to identify high-risk individuals could enable healthcare providers to more effectively tailor antiplatelet therapy, thereby reducing the risk of major adverse cardiovascular events. Integrating non-coding RNA analysis into clinical practice could help identify patients who require alternative or additional treatments, ultimately improving the overall management of cardiovascular and cerebrovascular diseases.

For ticagrelor, despite its proven efficacy in treating cardiovascular diseases, some patients may still develop resistance. Research indicates that non-coding RNAs, such as miR-126-3p and circFAM13B, may serve as potential biomarkers for predicting ticagrelor resistance and associated adverse ischemic events. Although the exact mechanisms of non-coding RNAs in ticagrelor resistance are still not fully understood, their potential in personalized treatment strategies is evident. Furthermore, prasugrel also enhances its antiplatelet effect by regulating miRNA levels, further confirming the crucial role of non-coding RNAs in modulating drug efficacy. In-depth investigation of these molecular mechanisms could provide new insights for optimizing clinical strategies and improving patient treatment responses and outcomes.

Although aspirin and clopidogrel remain cornerstone therapies for cardiovascular and cerebrovascular disease prevention, their drug resistance issues and potential adverse effects warrant significant clinical attention. From a pharmacoeconomic perspective, while clopidogrel demonstrates cost-effectiveness advantages and remains the most widely prescribed antiplatelet agent (van den Broek et al., 2022; Morris et al., 2022), substantial evidence indicates that ticagrelor shows superior efficacy in reducing composite endpoints of cardiovascular death, myocardial infarction, or stroke compared to clopidogrel (Wallentin et al., 2009), whereas prasugrel significantly decreases ischemic events (Wiviott et al., 2007; Ruff et al., 2012). Comprehensive analysis of clinical data reveals that both prasugrel and ticagrelor exhibit markedly better therapeutic outcomes than clopidogrel (Kumar et al., 2023; Orban et al., 2021; Schnorbus et al., 2020). Notably, for NSTE-ACS patients aged ≥70 years, clopidogrel remains the safer option due to its reduced bleeding risk (Gimbel et al., 2020). For clopidogrel non-responders, we recommend non-coding RNA profiling (e.g., miR-223) to guide clinical decisions, with ticagrelor or prasugrel serving as preferred alternatives given their efficacy advantages and lower resistance rates. Studies indicate that patients exhibiting miRNA-26a upregulation should consider switching to ticagrelor or prasugrel (Giantini et al., 2022). From a translational medicine perspective, future development should focus on targeted therapies modulating ncRNA networks (e.g., the miR-223-P2Y12 signaling axis) to precisely regulate platelet activation pathways and improve clinical outcomes. Regarding aspirin, while this low-cost conventional drug remains first-line therapy for cerebrovascular diseases, meta-analyses demonstrate that cilostazol offers multiple advantages in secondary stroke prevention: reduced composite vascular events, lower bleeding incidence (Dinicolantonio et al., 2013), and significantly better efficacy in preventing post-stroke vascular events (Kamal et al., 2011). Particularly, its combination with clopidogrel further decreases recurrent ischemic stroke risk (Hoshino et al., 2021). Therefore, for patients with aspirin treatment failure, in addition to considering switching to cilostazol, novel therapeutic strategies targeting pathways such as the H19/8-iso-PGF2α axis could be developed.

Although the association between non-coding RNAs and antiplatelet drug resistance has been confirmed by multiple studies, current evidence still exhibits significant limitations: Firstly, as shown in our article’s tables, most studies suffer from inadequate sample sizes and lack multicenter validation; secondly, circRNA-related research remains in its preliminary stages, requiring large-scale cohort studies to verify its clinical translational value; furthermore, miRNA expression profiles reported by different research teams demonstrate marked heterogeneity, which may stem from sample integrity and degradation issues during processing or be closely related to selection biases in library preparation methods (Ludwig et al., 2018; Lopez et al., 2015; Loudig et al., 2025). While epigenetic biomarkers show promising predictive potential, the sensitivity of current detection technologies still fails to meet clinical application requirements (Pirritano et al., 2018). Pharmacoeconomic evaluations indicate that personalized dosing strategies based on CYP2C19 genotypes offer significant cost-effectiveness advantages (Carroll et al., 2024), whereas the high costs associated with high-throughput sequencing technologies underscore the need for future research to focus on developing more economically viable detection platforms. Future studies should establish a clinical translation pathway for ncRNA research findings by: 1) implementing a three-tier detection system (CYP2C19 initial screening → ncRNA rapid panel secondary screening → sequencing confirmation); 2) developing CRISPR-Cas13a-based POCT devices; and 3) formulating biomarker-guided treatment decision matrices (e.g., switching to ticagrelor for patients with high miR-223 expression).

**FIGURE 3:**
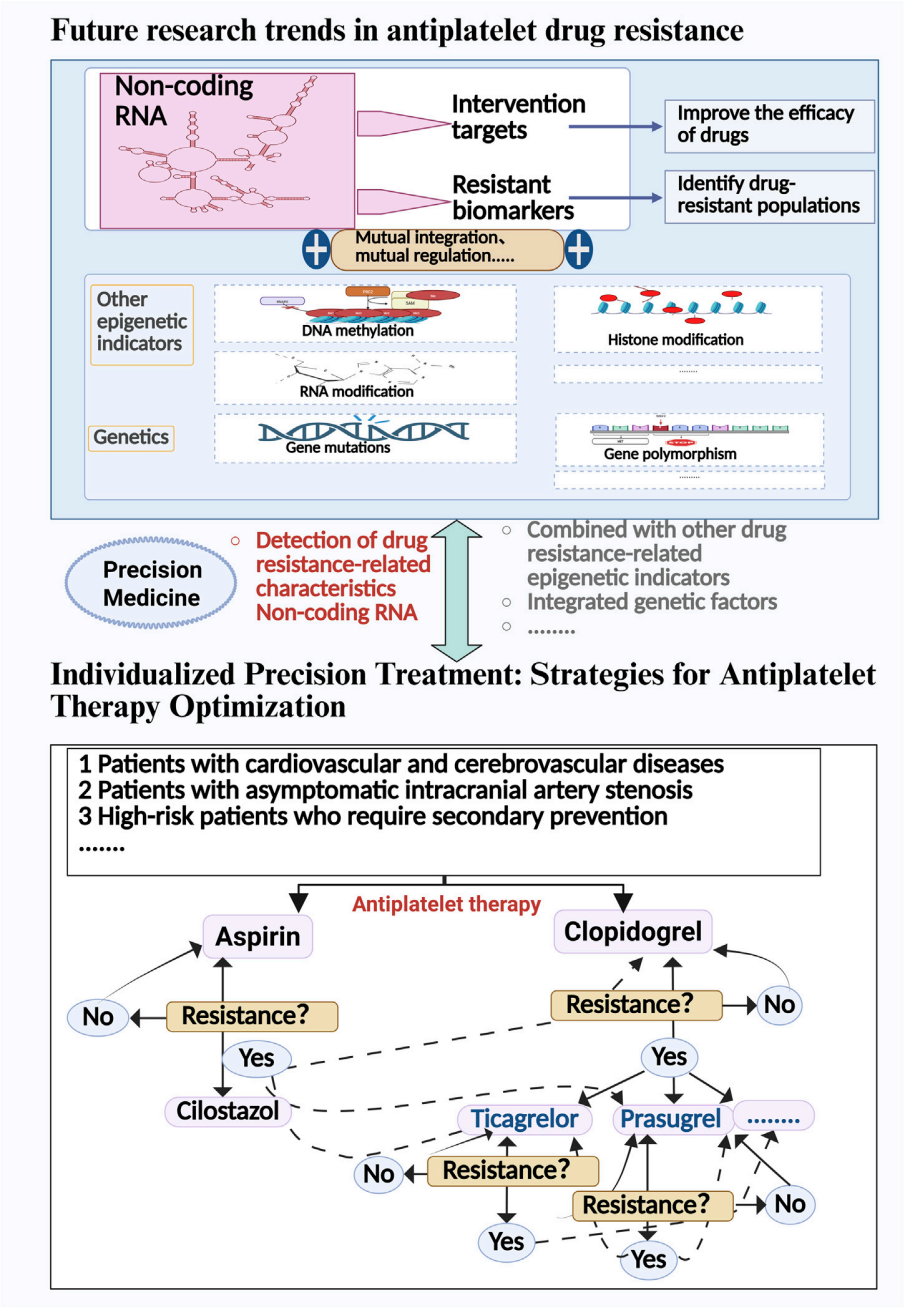
Possible targets and drug adjustment strategies for antiplatelet drug resistance.

Currently, bioinformatics tools, such as miRBase, MiRNA-BD, and CRAFT databases, can help gain a deeper understanding of the specific roles of non-coding RNAs in antiplatelet drug resistance and assist in predicting target genes (Kozomara et al., 2019; Lin et al., 2018; Dal Molin et al., 2022). However, the application of existing databases also presents significant limitations: many microRNA entries may lack precise tissue-specific origin or expression information, which could lead to misinterpretations in disease diagnosis or gene regulation studies (Singh, 2017; de Amo et al., 2022). In the past, there has been considerable genetic research on antiplatelet drug resistance, with some findings already applied in clinical practice (Pereira et al., 2019; Carroll et al., 2024; Feher et al., 2009). However, some patients continue to exhibit resistance. Additionally, the field of epigenetics, such as non-coding RNAs and DNA methylation, is also associated with antiplatelet drug resistance (Yang et al., 2020; Li et al., 2017). Therefore, future research urgently needs to address three key challenges: first, establishing standardized ncRNA detection protocols to resolve inter-laboratory reproducibility issues; second, enhancing the reliability of findings by expanding sample sizes and conducting multicenter collaborative studies; and third, developing integrated multi-omics prediction models that incorporate genomic, epigenomic, and clinical indicators. Figure 3 illustrates the potential targets of antiplatelet drug resistance and the strategies for drug modulation.

In conclusion, non-coding RNAs play a crucial role in the study of antiplatelet drug resistance, and the importance of RNA modifications in gene expression regulation cannot be overlooked. Future research should place greater emphasis on epigenetics, particularly exploring the potential of non-coding RNAs as biomarkers and therapeutic targets. Precision antiplatelet drugs offer new hope for resistance management and personalized treatment, while resistance testing based on the integration of epigenetic and genetic markers will be a key focus of future research.
